# Light-cured polymer electrodes for non-invasive EEG recordings

**DOI:** 10.1038/s41598-018-32304-6

**Published:** 2018-09-19

**Authors:** Nora Vanessa de Camp, Gerhard Kalinka, Jürgen Bergeler

**Affiliations:** 1 Institute of Animal Welfare, Animal Behavior and Laboratory Animal Science, Free University, Berlin, Germany; 2 Institute of Biology, Behavioral Physiology, Humboldt-University, Berlin, Germany; 3 Institute of Physiology, Medical Center of the Johannes Gutenberg University, Mainz, Germany; 4 Mechanics of Polymers, Bundesanstalt für Materialforschung- und prüfung (BAM) 5.3, Berlin, Germany

## Abstract

We invented the first non-metallic, self-adhesive and dry biosignalling electrode. The PEDOT polymer electrode changes its aggregate state and conductivity by a light curing procedure. The electrode can be applied as a gel underneath hair without shaving. With the aid of blue light, the electrode can be hardened within a few seconds at the desired location on the scalp. The cured polymer electrode is highly conductive and can be applied on a very small location. Unlike other EEG electrodes, our electrode does not lose conductivity upon drying. Furthermore, our electrode strongly bonds to skin and does not require any additional adhesive. Short circuits due to an outflow of gel are prevented with this technique. Therefore, the PEDOT polymer electrode is extremely well suited for applications that, up to now, have been challenging, such as non-invasive EEG recordings from awake and freely moving animals, EEG recordings from preterm babies in the neonatal intensive care unit or long-term recordings in the case of sleep monitoring or epilepsy diagnostics. We addressed two technical questions in this work. First, is the EEG recorded with polymer electrodes comparable to a standard EEG? Second, is it possible to record full-band EEGs with our electrodes?

## Introduction

Though non-invasive clinical electroencephalography (EEG) is a well-established method, technical limitations preclude its use in a wide area of possible applications. EEG amplifiers have seen enormous improvement regarding their input resistance, signal-to-noise ratio and degree of miniaturization^1,2^. In contrast, commonly used electrodes have not seen the same improvement. Several factors make the application of conventional electrodes challenging. The gold standard is the Ag/AgCl (silver/silver chloride) cup electrode, which must be filled with a conductive gel to achieve low-impedance contact with skin. These electrodes are rather bulky, and it is impossible to sleep on such electrodes, without causing pressure marks and pain. This makes their utilization in the neonatal intensive care unit, for sleep diagnostics and for long-term recordings difficult^3,4^. Furthermore, these electrodes can only be placed with the necessary stability if they are integrated into a head cap. Head caps are not recommended for younger children and are generally not well accepted^3^. Other solutions are disposable, self-adhering Ag/AgCl electrodes based on hydrogel^5^ or textile electrodes^4^. These electrodes are larger than the aforementioned cup electrodes. The larger surface area of these electrodes is necessary to ensure adequate adhesion to skin and low contact resistance. Though these electrodes are more flexible, most often shaving is required because hair impairs the necessary skin contact. Both electrode types described so far have one common impairment: they can lose functionality upon drying.

This flaw has driven the development of dry electrodes^6^. Dry electrodes, however, must be applied with pressure to the skin. Due to their rigid material characteristics, it is difficult to achieve stable contact to the skin, especially in combination with movement. A technical limitation is their strong capacitive behaviour, which makes the recording of very slow brain activity (delta band) impossible. The EEG of preterm babies is characterized by very slow delta oscillations, which can only be recorded with DC coupled amplifiers^7^. Therefore, full-band EEG (fbEEG) is an emerging standard in the neonatal intensive care unit^8^. Only electrodes with non-polarizing characteristics can be used for this purpose. In a systematic study with different metal electrodes, a sintered silver/silver chloride electrode in combination with a high-chloride electrode gel was best suited for full-band EEG recordings^9^.

Therefore, dry capacitive electrodes are generally not used in the neonatal intensive care unit and for stroke prevention or diagnosis.

Due to the technical limitations mentioned above, non-invasive EEG recordings are nearly impossible in the case of freely moving, awake animals. For scientific purposes, electrodes are implanted^10^. Most animals with implanted electrodes must be kept in isolation to protect the external parts of these devices, especially the connectors and pins, and to protect the animal from injury as consequence of the manipulation of external components. Currently, some attempts have been made to use non-invasive EEG electrodes for scientific purposes to strengthen translational research^11^. Nevertheless, these systems are not applicable to freely moving animals.

During veterinary care, animals are sedated, which in turn makes diagnosis difficult^12^. In a recent study, subdermal electrodes were used for epilepsy diagnostic in unsedated dogs^13^. The authors of this study report poor recording quality due to frequent artefacts of diverse origin, but they still report the general usefulness of the technique for diagnostic purposes.

The conducting part of all electrodes for non-invasive EEG recordings reported as yet is a metal, most often silver or gold. Over the past few years, a large array of non-metallic conducting materials have been developed^14^.

Poly(3,4-ethylenedioxythiophene) (PEDOT) is a conductive polymer used for the production of solar cells^15^ but is also a promising candidate for medical biosensors^16^. PEDOT itself is non-toxic^17^. Furthermore, surface coatings with poly(3,4-ethylenedioxythiophene) polystyrene sulfonate (PEDOT:PSS) have been shown to reduce gliosis around implanted neural interfaces^18^. Though PEDOT has been examined as an electrode material for neuroscience^19^, these attempts are restricted to surface coating. One report attempted to control the geometry of PEDOT through its deposition in titania nanotubes^20^. To the best of our knowledge, no formulation of PEDOT currently exists in which its conductive properties can be retained in a shapeable material.

**Figure 1:**
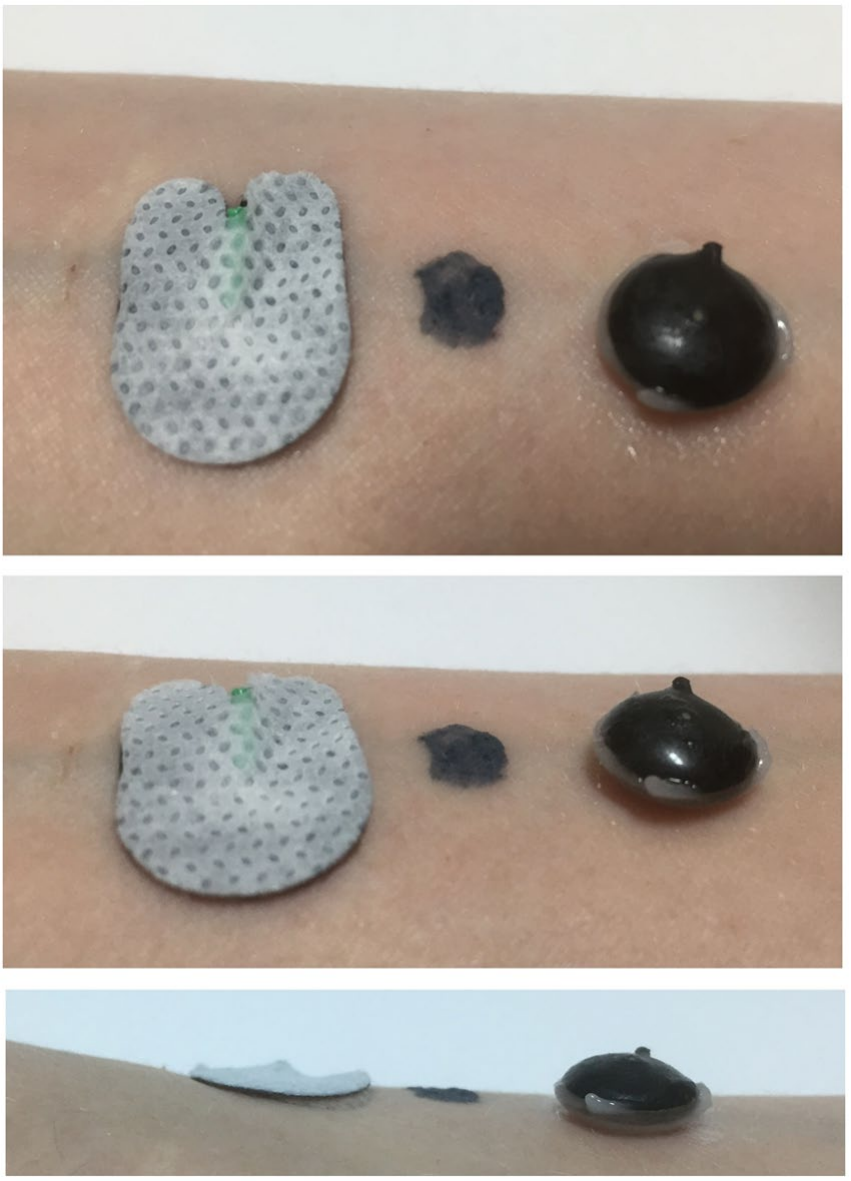
Three types of EEG electrodes on human skin. On the left, a relatively flat hydrogel electrode, the PEDOT polymer electrode (4 mm diameter) in the middle and on the right side a Ag/AgCl cup electrode with conductive gel (photograph created by Jürgen Bergeler).

Below, we describe a formulation of PEDOT in combination with another polymer. This gel electrode is initially fluid and therefore can be smoothly applied to the skin on a very small surface area and independent of hair growth. The electrode changes its aggregate state by the application of blue light for a few seconds. In the dry state, the electrode is highly conductive and self-adheres to the skin (Fig. 1). The close and flexible skin contact gives stable recordings, even in the case of freely moving animals. The electrodes can be easily removed without water or pain, even if applied under hair.

## Results

### EEG recordings from freely moving piglets

To examine relative deviations in the EEG power between bands with both types of electrode, we calculated the normalized difference between the EEG band power recorded with PEDOT polymer electrodes and the band power recorded with commercially available Ag/AgCl hydrogel electrodes. To examine possible asymmetries in the spectral power density, the normalized differences were compared between bands. If the EEG bands are represented relatively well by both types of electrodes, the normalized difference between bands should not be different. No statistically significant deviations in the spectral power differences between EEG bands were observed (Tables 1 and 2).

**Table 1:** Mean spectral power for both types of electrodes used for EEG recordings in a pigpen (PEDOT polymer and self-adhesive hydrogel electrodes) and values for the normalized difference as well as standard deviation.

Frequency Band	Mean Power [µV^2^/ms]	Mean normalized difference	Standard deviation of the normalized difference
delta	Hydrogel: 1.18 PEDOT: 1.17	0.15	0.09
theta	Hydrogel: 2.74 × 10^−5^ PEDOT: 1.33 × 10^−4^	0.38	0.32
alpha	Hydrogel: 5.92 × 10^−5^ PEDOT: 2.83 × 10^−4^	0.38	0.32
beta	Hydrogel: 7.76 × 10^−5^ PEDOT: 3.74 × 10^−4^	0.39	0.32
gamma	Hydrogel: 1.14 × 10^−4^ PEDOT: 5.53 × 10^−4^	0.41	0.32
high-gamma	Hydrogel: 3.00 × 10^−5^ PEDOT: 1.75 × 10^−4^	0.45	0.35

**Table 2:** Results of the multiple comparison test (Matlab) based on the stats matrix of ANOVA (Matlab 2016b) following a Lilliefors test (Matlab 2016b), which confirmed that the data are normally distributed.

Group1	Group2	Lower CI	Dif Groups	Upper CI	p-value
delta	theta	−0.88	−0.23	0.41	0.76
delta	alpha	−0.87	−0.23	0.41	0.77
delta	beta	−0.89	−0.24	0.40	0.73
delta	gamma	−0.91	−0.26	0.38	0.65
delta	hgamma	−0.95	−0.31	0.34	0.50
theta	alpha	−0.64	0.00	0.65	1.00
theta	beta	−0.65	−0.01	0.63	1.00
theta	gamma	−0.68	−0.03	0.61	1.00
theta	hgamma	−0.72	−0.08	0.57	1.00
alpha	beta	−0.66	−0.01	0.63	1.00
alpha	gamma	−0.68	−0.03	0.61	1.00
alpha	hgamma	−0.72	−0.08	0.57	1.00
beta	gamma	−0.66	−0.02	0.62	1.00
beta	hgamma	−0.71	−0.06	0.58	1.00
gamma	hgamma	−0.69	−0.04	0.60	1.00

If the confidence interval (CI) does not contain zero, the difference is statistically significant. No statistically significant difference was observed in the EEG band power deviation between the EEG bands for both types of electrodes tested.

**Figure 2:**
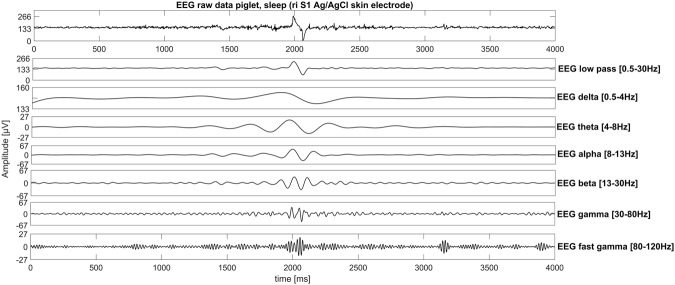
Sleep EEG recording from freely moving piglets. A K-Komplex-like structure recorded with a self-adhesive hydrogel electrode on the basis of Ag/AgCl in the right somatosensory cortex region.

**Figure 3:**
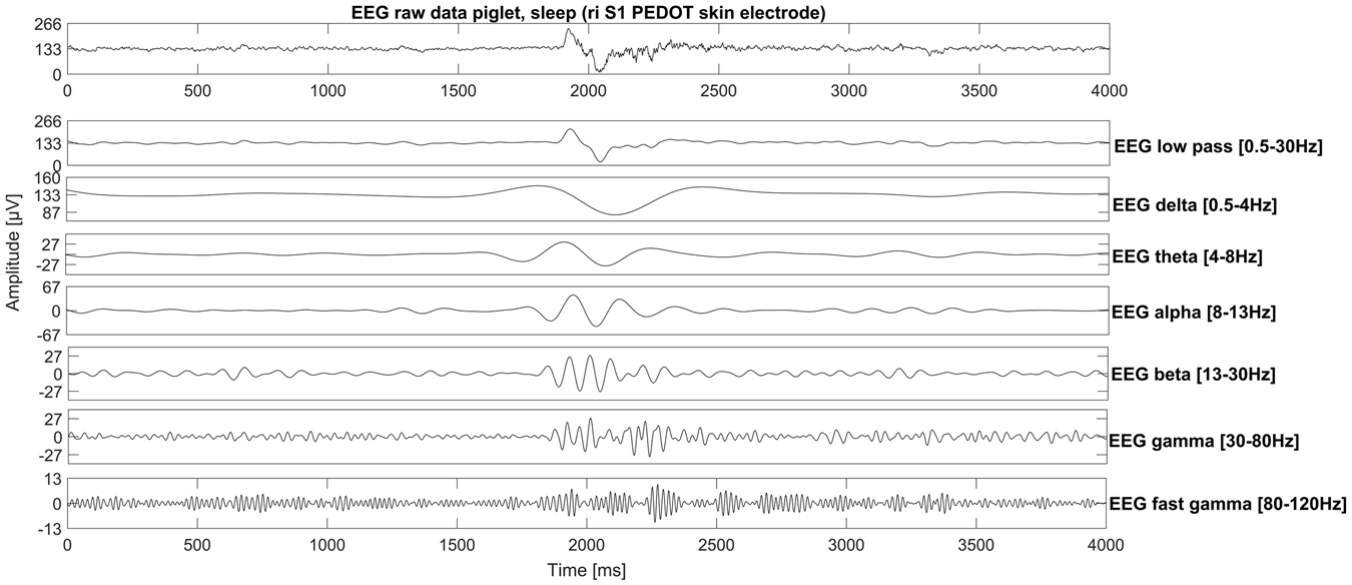
Sleep EEG recording from freely moving piglets. A K-Komplex-like structure recorded with a self-adhesive PEDOT polymer electrode in the right somatosensory cortex region.

With both types of electrodes, we recorded typical patterns such as spindle bursts, spontaneously occurring delta brushes or k-complex-like events during drowsiness and sleep for freely moving piglets in a pigpen (Figs 2 and 3).

### Impedance measurement

**Figure 4:**
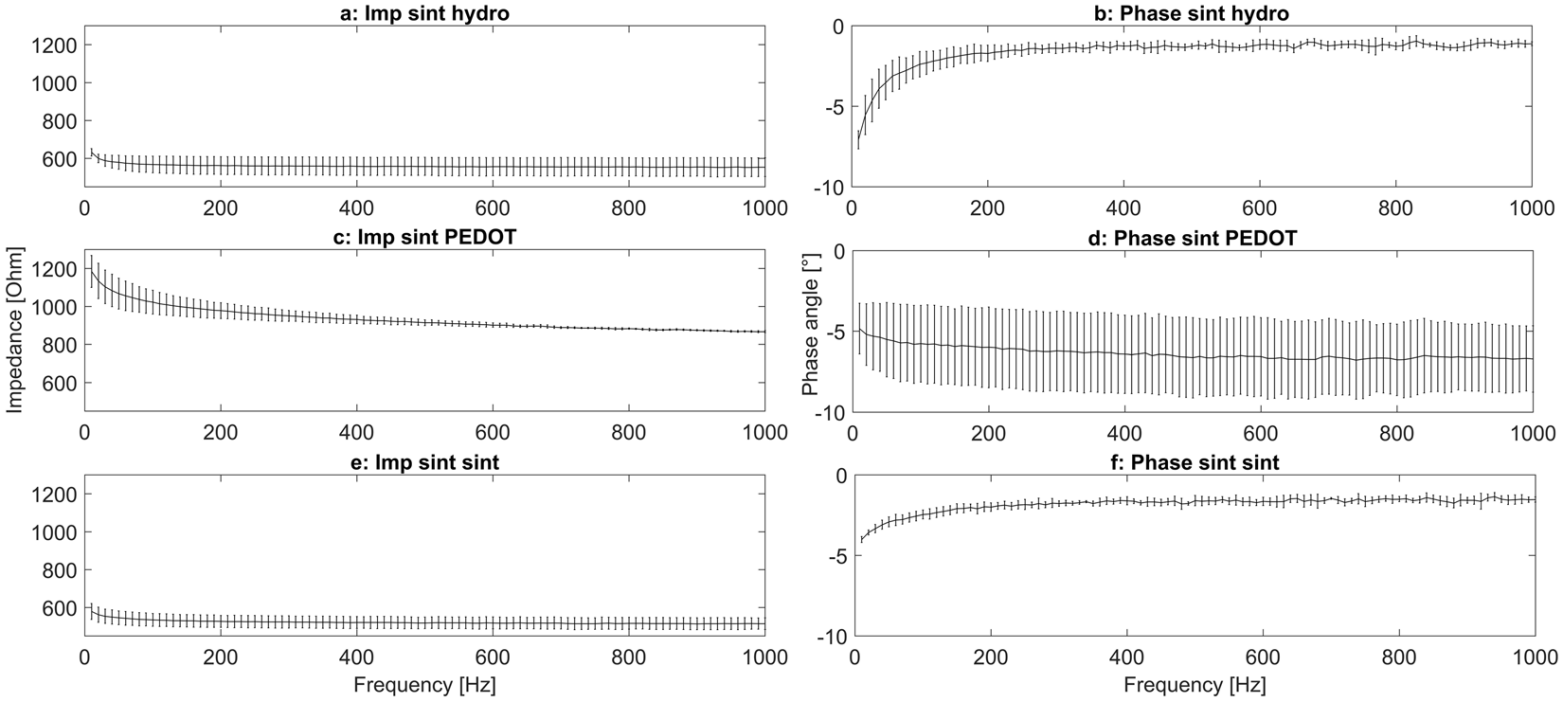
Impedance and phase angle of the PEDOT polymer and Ag/AgCl electrodes on agar plates. Measurement of electrodes on the top of an agar plate with a physiological sodium chloride concentration. The gold standard is the measurement of two Ag/AgCl electrodes (sint) against each other (**a** and **b**, always 10 cm between electrodes on the plate). Additionally, a self-adhesive hydrogel electrode was measured against Ag/AgCl (sint hydrogel, **c** and **d**). The PEDOT polymer electrode had a surface of 2 × 3 mm, approximately half the surface of the Ag/AgCl electrodes. The impedance of the PEDOT polymer electrodes (**c**) is slightly higher than the other electrodes (**a**,**e**). The absolute shift in the phase angle is comparable between electrodes, but the PEDOT polymer electrodes show a higher standard deviation (**d**, error bars) than the Ag/AgCl (**f**) and hydrogel electrodes (**b**).

The impedance was measured in a range from 10 Hz to 1000 Hz and results in values between 1.2–0.8 kOhm for the PEDOT polymer electrodes (Fig. 4c). The silver electrodes (Fig. 4a,e) had an impedance of 0.5 kOhm. Phase angle changes during the impedance measurement can be observed in all electrodes. The silver hydrogel electrodes (hydro) had a change of approximately 5° during the impedance measurement (Fig. 4b). The sintered silver electrode (sint) had a change of approximately 3° (Fig. 4f). In contrast, the PEDOT polymer electrode had a change of approximately 2° but with a relatively high standard deviation (error bars in Fig. 4d).

**Figure 5:**
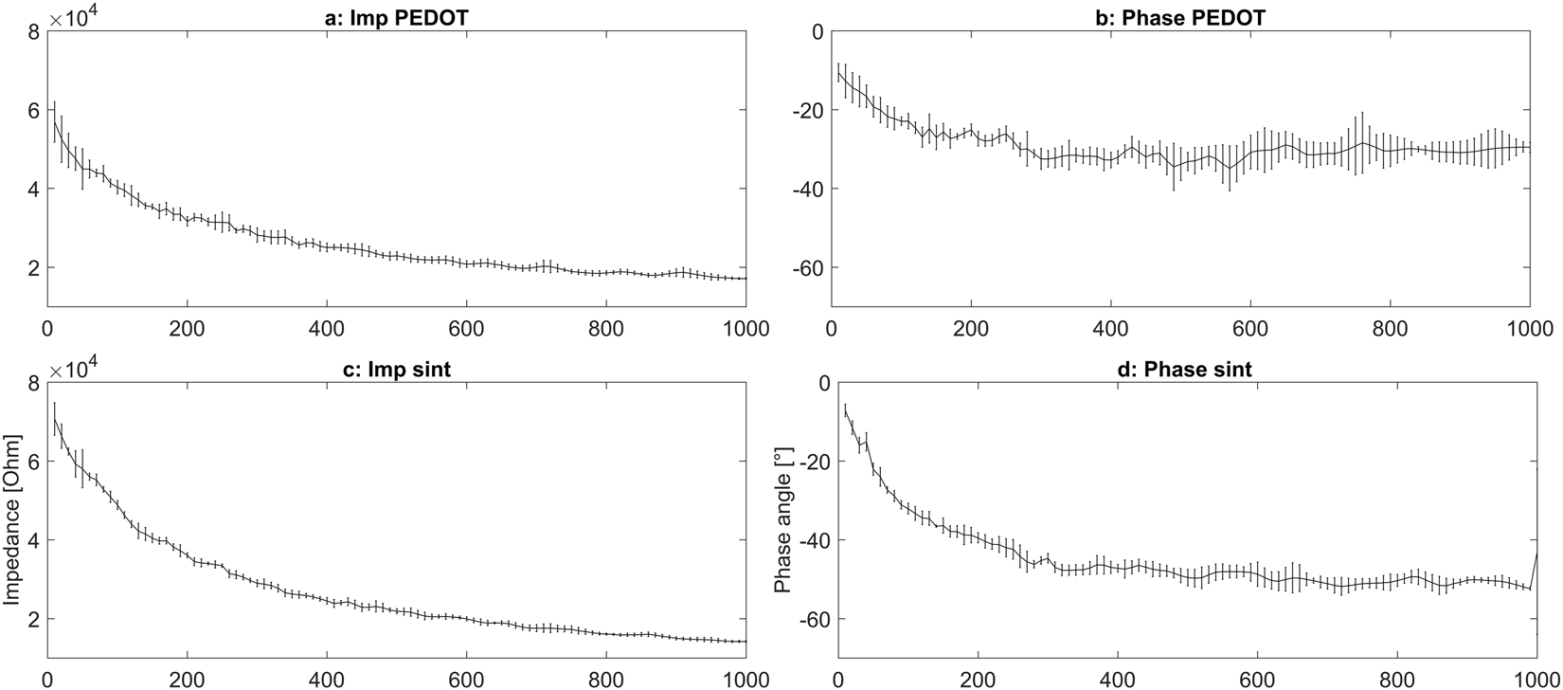
Impedance of the PEDOT polymer electrode on skin (hand). The impedance was measured from 10 to 1000 Hz on skin against a second PEDOT polymer electrode 10 cm away. The same measurement with exactly the same positions and distance was performed with Ag/AgCl electrodes. Both types of electrodes show similar results.

Furthermore, we measured the impedance on skin (hand). In contrast to the measurement on agarose, the PEDOT polymer electrodes showed slightly lower impedance (Fig. 5a) and lower changes in the phase angle (Fig. 5b) than the sintered silver/silver chloride electrodes with an electrode gel (Fig. 5c,d) when measured on skin. Both electrodes showed similar standard deviations.

**Figure 6:**
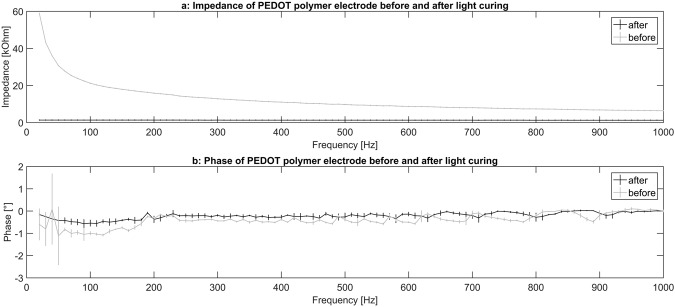
Impedance and phase angle of the PEDOT gel before and after the application of blue light. The impedance is reduced from about 60 kOhm to about 1 kOhm after the application of blue light (upper panel, dark trace after the application of blue light). After the light curing procedure a reduced variance of the phase angle can be seen (lower panel, dark trace after the application of blue light). Error bars show standard deviation.

Additionally, the impedance (Fig. 6a) and phase angle (Fig. 6b) of the PEDOT polymer electrode were measured before and after the light curing procedure (n = 4). In this case, the impedance was relatively high before light curing [before curing: 10 Hz = 59 kOhm (standard deviation (SD) 0.2), 100 Hz = 20 kOhm (SD 0.2), 500 Hz = 11 kOhm (SD 0.2); after curing: 10 Hz = 1.4 kOhm (SD 0.2), 100 Hz = 1.4 kOhm (SD 0.2), 500 Hz = 1.4 kOhm (SD 0.2)], and the phase angle varied stronger before curing [before curing: 10 Hz = −0.6° (SD 0.7), 100 Hz = −1° (SD 0.03), 500 Hz = −0.5° (SD 0.04); after curing: 10 Hz = −0.2° (SD 0.05), 100 Hz = −0.6° (SD 0.1), 500 Hz = −0.2° (SD 0.08)] (Fig. 6).

### Drift during full-band DC EEG recording

Furthermore, we examined the stability of the PEDOT polymer electrode under full-band DC EEG conditions. For this purpose, we took a recording with standard silver electrodes in combination with a high-chloride gel (1020 electrode cream) and nearby PEDOT polymer electrodes. The difference in DC drift is not statistically significant between PEDOT polymer electrodes and standard Ag/AgCl electrodes in combination with a gel (alpha = 0.01, p-value 0.4452, Wilcoxon Rank-Sum Test, Matlab 2016b).

### Mechanical measurements

**Figure 7:**
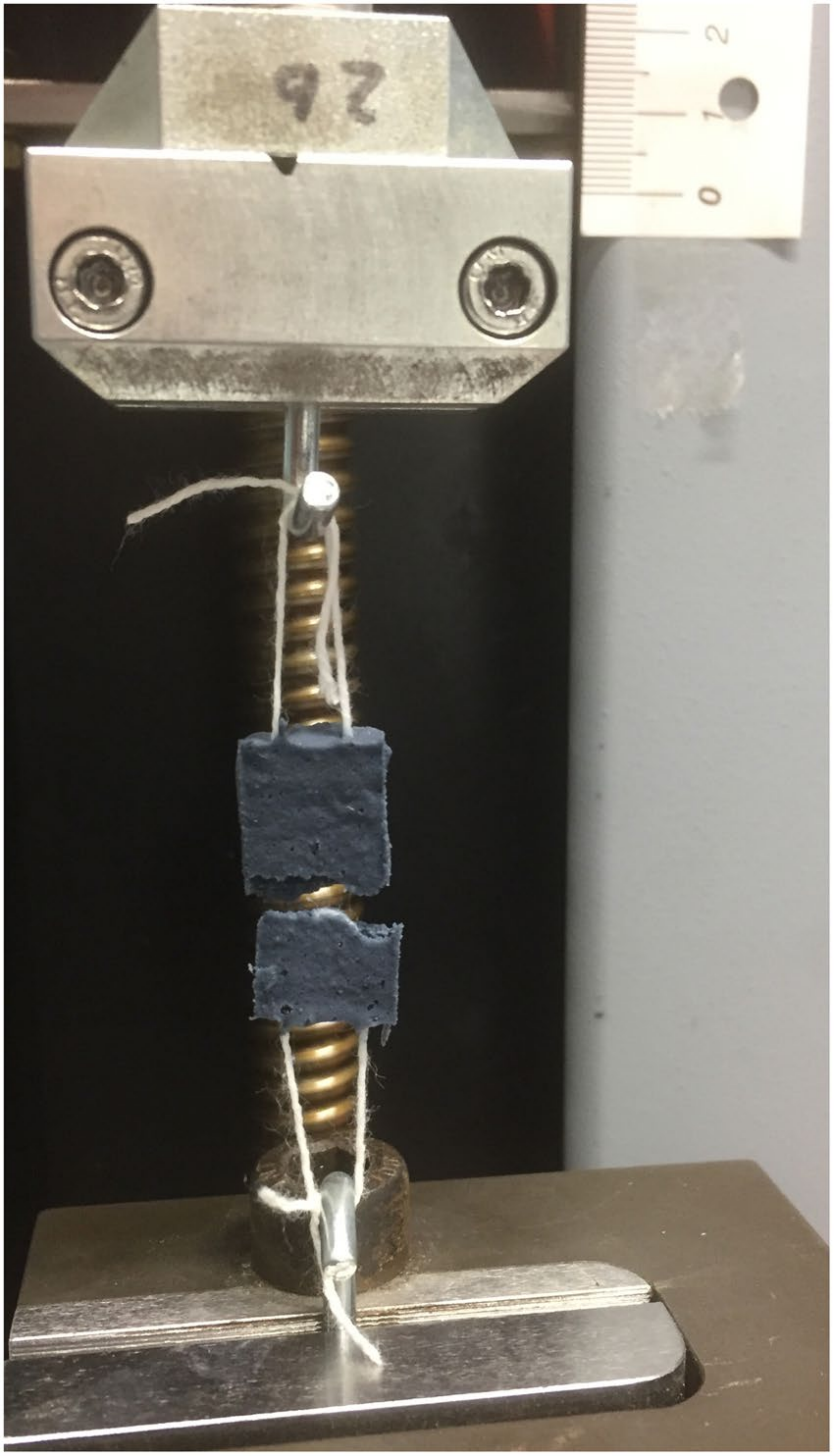
Photograph of the failure during mechanical testing of the PEDOT polymer electrode. The strings were embedded inside the sample and served as connector to the extensometer.

**Figure 8:**
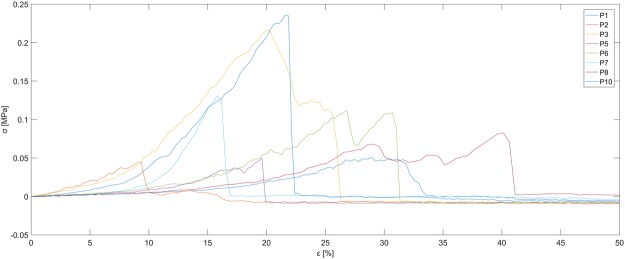
Stress-Strain measurement. 10 equally sized samples of the PEDOT Polymer electrode material (10 × 2 × 20 mm) were measured until a failure of the material occurred (steep drop of sigma). On the x-axis, the normalized elongation of material is shown (epsilon), on the y-axis the force per area is shown (sigma [MPa]).

**Figure 9:**
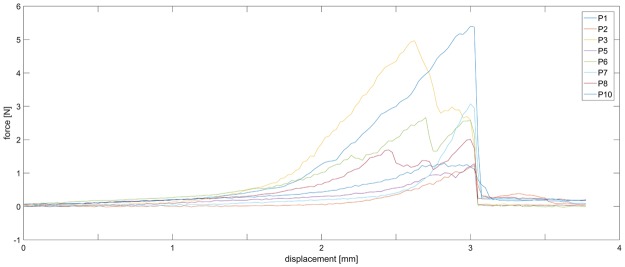
Force measurement. 10 equally sized samples of the PEDOT Polymer electrode material (10 × 2 × 20 mm) were measured until a break of the material occurred. To compare the relationship between force and elongation, the slopes of individual measurements are aligned at the point of failure (a sudden drop of force). A relatively high degree of variation is visible, which may be due to the curing procedure with a handheld blue LED light. Two samples were discarded due to incomplete curing (Nr. 4 and Nr. 9).

The material is soft, but has a brittle failure characteristic. The failure takes place in the middle of the sample after an elongation between 10 and 40% displacement (Fig. 7). The stress range is between 0.05 to 0.2 MPa (Fig. 8). In order to allow a better comparison, we shifted the data along the displacement axis to get the same displacement of failure (Fig. 9). A high variance of force and elongation can be observed. Two samples (Nr. 4 and Nr. 9) were discarded due to their obviously incomplete curing which became apparent after the distortion.

## Discussion

Our PEDOT polymer electrode is the first non-metallic EEG electrode that changes its aggregate state by a light curing procedure within a few seconds. With this electrode, we can obtain several important prerequisites for stable long-term EEG recording. First, our electrode does not lose functionality upon drying, as is the case for all standard electrodes applied with gel. In addition, our electrode is self-adhesive, which is not the case for the other dry electrodes described so far. The change in aggregate state is important for the application of very small amounts of the fluid electrode gel underneath hair without the necessity of shaving. After curing directly on the skin with blue light, the electrode is highly conductive and stable but still nearly invisible. This makes the electrode ideal for everyday use, for example, in the case of epilepsy diagnostics or stroke prevention. The miniaturized flat and smooth characteristics of the electrodes make them extremely comfortable for the user (Fig. 1). This is important to prevent pressure marks in the case of young children, those in the neonatal intensive care unit or for sleep diagnostics. The electrode can easily be removed by the application of pressure from two sides. We performed mechanical measurements with this new electrode material (Figs 7–9). The high degree of variance may be due to the curing with a dental handheld blue LED light. A sample size of 10 × 2 × 20 mm was necessary for the experimental design. For application on the human skin, the overall size of the electrodes is substantially smaller (Fig. 1). The low forces required for material destruction ensure the prevention of skin injury. On the other hand, the strength is high enough for proper use and adhesion on the skin. Furthermore, these material characteristics enable a pain-free removal of the PEDOT polymer electrodes after use. Residues, if present, can easily be brushed out. Other applications of this new material include electrocardiography, electromyography or electroenterography. The recording characteristics are very similar to those of standard silver/silver chloride electrodes in combination with a high-chloride gel, as shown by our test recordings in the pigpen (Figs 2 and 3). The performance of the PEDOT polymer electrode depends on close contact to the particular surface. In the case of agarose, as used in our impedance measurements (Fig. 4), the PEDOT polymer electrode has loose contact, which may be the reason for the relatively high impedance and high standard deviation for the phase angle. Therefore, we also tested the impedance on human skin (Fig. 5). In this case, the impedance values and corresponding phase angles measured with the PEDOT polymer electrodes are very similar to those with sintered Ag/AgCl electrodes. Furthermore, the PEDOT polymer electrode has similar DC drift characteristics to the silver/silver chloride electrode, as shown by a long-term full-band DC EEG recording with a human test person. This makes the use of the electrode in the neonatal intensive care unit extremely interesting, as slow oscillations play an important role during preterm cortical development. Beyond electrophysiological applications, the material is likely well suited for 3D-printed complex material combinations of insulating and conductive materials.

For the Future, we are working on metal free, soft and flexible cables as well as application systems with integrated blue light LED for the fast and precise handling of the PEDOT polymer electrode.

## Materials and Methods

### Animal Experiments

We took EEG recordings of 6 newborn piglets. All procedures were approved by the local ethics committee (#23177-07/G10-1-010/G 15-15-011, Landesuntersuchungsamt Rhineland-Palatinate, Germany) and followed European and German national regulations (European Communities Council Directive, 86/609/ECC; Tierschutzgesetz).

All animal procedures were performed in accordance with the [Medical Center of the Johannes Gutenberg University Mainz] animal care committee’s regulations. Piglets were calmed down by wrapping them in a towel, and no anaesthesia was used. The electrode fixation procedure lasted approximately 5–10 minutes. The PEDOT polymer electrode was fixed as described below. Afterwards, the pin of the electrode cable was connected to a telemetric 1-channel EEG system. The system had dimensions of approximately 1 × 2 cm and weighed 10 g. The system was fixed with a non-toxic body silicone (body double fast, Smooth-On Inc., USA) on top of the piglet’s head, above the electrode.

### EEG recordings

Disposable adhesive-surface silver/silver chloride electrodes (Spes Medica S.r.l., Genova, Italy) were placed above cerebellum (between the ears) as the ground, above the nose as the reference and between eye and ear to record from the right somatosensory cortex region (parietal position). Before fixation of the electrode, the location was shaved (in the case of hydrogel electrodes), and the skin was cleaned with an abrasive cream (Abralyt HiCl, Easycap GmbH, Herrsching, Germany) to remove dead skin cells and to achieve a lower impedance. We used a 1:1 mixture of PEDOT:PSS (Heraeus, Clevios SV3) and the dental cement formulation Tetric EvoFlow® (ivoclar vivadent) as light curing gel electrode for EEG recordings from freely moving piglets in the pigpen. The skin was cleansed of dirt and dead skin cells by an abrasive treatment (Abralyt, HiCl, Easycap, Germany). Afterwards, a silver wire was deinsulated at the tip and fixed at the desired position on the head. The PEDOT polymer electrode gel was placed on top of the insulated wire and cured with blue light within 20 seconds. For the curing, we used a dental blue light (Drs Light Clever DUAL, 400–490 nm, 1500 mW/cm^2^). The distance should be in the range of 0.5 to 1 cm. The gel can be applied underneath hair; therefore, shaving was only necessary for the control group with self-adhesive Ag/AgCl hydrogel electrodes. The data were recorded and sent by a telemetry unit (with an AC coupled amplifier, sampling rate 500 Hz)^21^. Only phases without artefacts were taken into account for analysis. The thickness of the PEDOT polymer electrodes is about 0.5 mm, the diameter is 5 mm with round shape.

### EEG analysis

We analysed the data with Matlab (2016b, Simulink) and with brainstorm^22^. The EEG raw data were filtered with digital Butterworth filters with a custom-written Matlab script. The filter was designed with a Butterworth function (n = 3rd order). We calculated the normalized cutoff frequency (Wn) for the delta [0–4 Hz], theta [4–8 Hz], alpha [8–13 Hz], beta [13–30 Hz], low-gamma [30–80 Hz] and high-gamma [80–120 Hz] EEG bands. Wn is a number between 0 and 1, where 1 corresponds to the Nyquist frequency, which is half the sampling rate (here, 250 Hz for down-sampled EEG data).

The numerator and denominator values (IIR filter), achieved with the function butter, were used with the Matlab function filtfilt to filter the EEG data. For the delta EEG band (0–4 Hz), a lowpass was used. We extracted all other EEG frequency bands with a bandpass filter design.

To find relative deviations of band power between electrodes, we calculated a normalized difference of band power.

### Statistics

We tested the data distribution with the Lilliefors test (Matlab 2016b, Simulink). Tests with more than two groups were performed with the non-parametric Kruskal Wallis test or ANOVA (in the case of normal distribution) and a subsequent multiple comparison test in order to achieve the exact statistical relations between groups. All tests were implemented in the Matlab statistics toolbox (2016b, Simulink). If two groups were compared, we used the non-parametric Wilcoxon Rank-Sum Test.

### Impedance measurement

We measured the impedance of the PEDOT polymer electrode with a Red Pitaya device (Impedance analyser module, Stem lab, Solkan, Slovenia). The PEDOT polymer electrode was placed on the skin and measured against a second PEDOT polymer electrode 10 cm away. At the same physical points, a silver/silver chloride electrode was measured against a second silver/silver chloride electrode (Ag/AgCl) in combination with gel (Ten-20, Weaver, USA). The impedance was measured from 10 to 1000 Hz on a logarithmic scale. Five measurements were taken for each set of electrodes. The measurements started 30 minutes after placement of the electrodes on the skin.

Furthermore, we measured the impedance of the PEDOT polymer, hydrogel and sintered Ag/AgCl electrodes against a Ag/AgCl reference electrode on an agar plate under physiologic concentrations with sodium chloride (3% agar in 150 mM NaCl solution). The distance between electrodes was 10 cm. The Ag/AgCl electrodes had dimensions of 5 mm diameter × 5 mm thickness (round shape), the hydrogel electrodes had an oval shape with dimensions of 10 × 8 mm and 2 mm thickness. The PEDOT polymer electrodes had a round shape with dimensions of 3 mm diameter × 2 mm thickness.

For the measurements before and after light curing we used an LCR-6100 (GW Instek, Taiwan) as impedance analyser. Furthermore we designed a setup with 2 cm PEDOT polymer electrode and 5 cm conductive path on each side. For a quadrupole impedance measurement we placed one Kelvin clamp on each conductive path (PEDOT surface) and measured from 10 to 1000 Hz.

### DC drift

To investigate the DC stability of the PEDOT polymer electrodes, we took one EEG measurement on a human test subject. For each recording position, we applied one PEDOT polymer electrode and one standard Ag/AgCl cup electrode in combination with a high-chloride gel (Ten-20, Weaver, USA) in close proximity. Data acquisition was achieved with a medically approved Neuroconn (Neuroprax) full-band DC EEG monitoring system (Neuroconn, Ilmenau, Germany). The recording took approximately 10 minutes per session. We used only electrodes that were characterized as good quality by the system. The Neuroconn quality assessment system relies on the signal-to-noise ratio. To prevent any influence on the running recording, no current was applied. We calculated the drift of 6 Ag/AgCl electrodes and 6 PEDOT polymer electrodes by determining the difference in voltage at the beginning and end of the recording session (10 minutes recording).

### Stress-Strain measurement

10 PEDOT polymer samples with a size of 10 × 2 × 20 mm were produced by using a polypropylene form. Before curing, a 10 cm long piece of polyethylene fiber (Nikol Weber KG, Selbitz, Germany) was inserted into each end of the sample as a slope. Each fiber was interloped and served as holder for the measurement in the extensometer. For Fig. 9 we cut the measured curves at the point of full elongation of the polypropylene fiber. At that point, the main part of the force affects the sample. We did one test measurement with the same extensometer and only the fiber. The fiber has an extremely steep rise of force (6 N for 0.4 mm displacement) in contrast to the PEDOT samples.

## Data Availability

Data are available on demand.
